# Re-examination of successful agers with lower biological than chronological age still after a 20-year follow-up period

**DOI:** 10.1186/s12877-023-03844-y

**Published:** 2023-03-07

**Authors:** Anna Viljanen, Marika Salminen, Kerttu Irjala, Päivi Korhonen, Tero Vahlberg, Matti Viitanen, Minna Löppönen, Laura Viikari

**Affiliations:** 1 Wellbeing Services County of Southwest Finland, Turku University Hospital, Domain of General Practice and Rehabilitation, Turku, Finland; 2 Department of Clinical Medicine, Faculty of Medicine, Unit of Geriatric Medicine, University of Turku and Turku City Hospital, Kunnallissairaalantie 20, 20700 Turku, Finland; 3 Wellbeing Services County of Southwest Finland, Turku University Hospital, Medical Domain, Geriatric Medicine, Turku, Finland; 4 Faculty of Medicine, Department of Clinical Medicine, Unit of General Practice, University of Turku and Turku University Hospital, 20014 Turku, Finland; 5 Faculty of Medicine, Department of Clinical Medicine, Unit of Clinical Chemistry, University of Turku and Turku University Hospital, 20521 Turku, Finland; 6 Faculty of Medicine, Department of Clinical Medicine, Unit of Biostatistics, University of Turku and Turku University Hospital, Turku, Finland; 7 Division of Clinical Geriatrics, Center for Alzheimer Research, Department of Neurobiology, Care Sciences and Society, Karolinska Institutet and Karolinska University Hospital, Huddinge, Stockholm, Sweden

**Keywords:** Personal biological age, Resilience, Successful ageing, Satisfaction with life

## Abstract

**Background:**

Successful ageing is the term often used for depicting exceptional ageing but a uniform definition is lacking. The aim was to re-examine and describe the successful agers living at home at the age of 84 years or over after a 20-year follow-up. The purpose was also to identify possible factors leading to their successful ageing.

**Methods:**

Successful ageing was defined as the ability to live at home without daily care. Data on the participants’ functional ability, objective health, self-rated health and satisfaction with life were gathered at baseline and after a 20-year follow-up period. A measurement of personal biological age (PBA) was established and the difference between the PBA and the chronological age (CA) was counted.

**Results:**

The participants’ mean age was 87.6 years (Standard deviation 2.5, range 84–96). All analyzed variables depicted poorer physical ability and subjective health at re-examination than at baseline. Still, 99% of the participants were at least moderately satisfied with their lives. The PBA at baseline was 6.5 years younger than CA, and at re-examination, the difference was even more pronounced at 10.5 years.

**Discussion:**

Even though the participants were chronologically older, had poorer physical ability and subjective health, they were still satisfied with their lives indicating possible psychological resilience. The difference between the PBA and CA was greater at re-examination than at baseline indicating that they were also biologically successful agers.

**Conclusions:**

Successful agers were satisfied with life despite hardships and had a lower biological than chronological age. Further research is needed to evaluate causality.

**Supplementary Information:**

The online version contains supplementary material available at 10.1186/s12877-023-03844-y.

## Background

Successful ageing (SA) is the term often used in research for depicting extraordinary or exceptional ageing in contrast to usual ageing. It has been defined in various ways but a common, unified definition is still lacking [1]. The traditional concept by Rowe and Kahn (1997) depicted SA as the absence of disease and disability, high cognitive and physical function and engagement with life [2]. Researchers nowadays often include also constructs such as social engagement, satisfaction with life, and independence [1]. SA does not necessarily mean the absence of disease, disability or frailty, but includes also psychological and social factors. However, in research, SA is still most often defined as the absence of disability [3].

SA has been investigated as the possible opposite of frailty and the two do have similarities that could imply a continuum [4]. However, when investigating the prevalence of frailty and SA, there were overlaps of the SA group with the frail and pre-frail groups opposing the idea of a continuum [4]. SA is not simply the same as robust.

The meaning of ageing in place (dwelling in the community) has been related to a sense of identity through independence and autonomy [5]. Also, social networks and the surroundings have been considered important resources for ageing well in place [5]. A study investigating near-centenarians and centenarians found that even though they had poor objective health, their subjective health was good [6]. This suggests that these survivors had high psychological (and perhaps also physical) resilience. Psychological resilience [7] refers to the person’s ability to adapt when facing a stressor, and physical resilience [8] to the ability of maintaining or recovering function after the stressful event [9]. Resilience has earlier been suggested to play a part in ageing successfully [10].

Frailty by the Frailty Index (FI) has also been proposed as the proxy measure of ageing [11], and a personal biological age (PBA) can be counted from the FI score by investigating the mean FI for each age in a study population and then comparing the FI score of an individual to the mean scores of the population to determine the PBA of that individual [12]. The individuals’ PBA can be significantly lower than their chronological age (CA) making them biologically younger than their years and as such, a successful ager.

Traditionally, assessing for instance risks for surgery has been done by the eyeball test, i.e. a clinician’s assessment of the patient’s frailty status, and thus their biological age, done before the operation by an end-of-bed evaluation [13]. The eyeball test has, however, been found to poorly correlate with the frailty status [14].

In our previous studies we found that frailty and poor subjective and objective health predicted mortality among community-dwelling older people [15, 16]. We also found that frailty, poor self-rated health (SRH) and self-reported inability to walk 400 m, as well as certain chronic conditions (dementia, mood or neurological disorders) and multimorbidity (defined as having five or more chronic conditions) predicted institutionalization in the same study population [17, 18]. In this study, our aim was to describe the successful agers, defined as a person still living at home without daily formal or informal care (absence of disability) at the chronological age of 84 years or older, and to investigate the changes from baseline to the end of a 20-year follow-up period in the same variables used in our previous studies. Also, as our definition of SA was the absence of disability/independence, we wanted to investigate whether these successful agers had other factors, such as good SRH or good satisfaction with life, that have earlier also been linked to ageing successfully [1]. In Finland, the criteria for receiving daily formal home care are strict and only met when a person needs help in activities of daily living (ADL). So to receive daily formal care is a clear indicator of disability.

## Methods

### Study population

This study is part of the longitudinal Lieto study, a clinical, epidemiological study of subjects aged 64 years or older. It was carried out in Lieto, a semi-industrialized rural municipality in South-Western Finland.

All residents born in 1933 or earlier living in Lieto on February 16th of 1998 (*n =* 1596; 666 men and 930 women, 12% of the population) were invited, in a random order, to participate in the study at baseline. Of those eligible, 63 died before the baseline examination and 273 refused or did not respond. Altogether 1260 (82%) subjects participated in the baseline examination between March 1998 and September 1999, 533 men and 727 women. The baseline examination is described elsewhere [19].

**Fig. 1:**
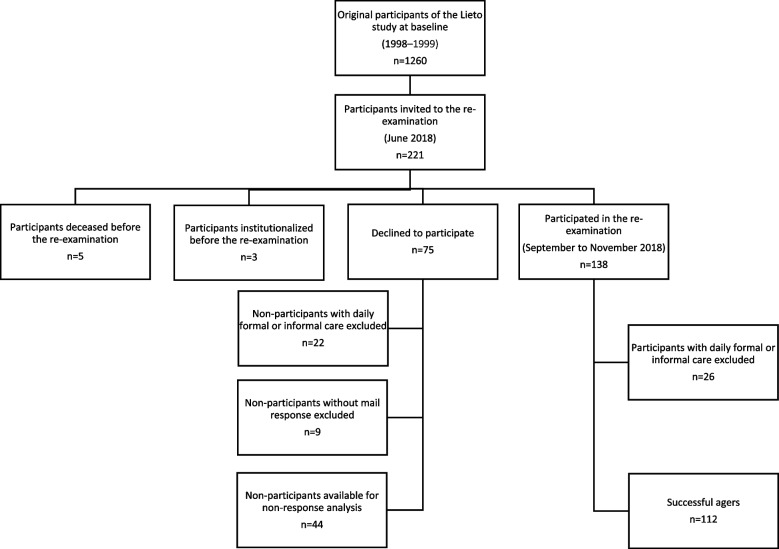
Flow chart of the study participants

For re-examination that took place between September and November of 2018, we invited the original Lieto study participants still living at home in the municipality of Lieto in June of 2018 (*n =* 221). Before the re-examination, five were deceased and three were institutionalized. Seventy-five subjects did not participate, leaving 138 participants for the re-examination. The aim of this study was to describe the true successful agers so the participants with need of formal or informal daily care were excluded from the analyses leaving 112 participants and 53 non-participants, of which 44 responded by mail and their responses were used in the non-response analysis. A flow-chart of the study is shown in Fig. 1.

### Mortality

Data from all participants who died before January 2017 were obtained from the official Finnish Cause of Death Registry using unique personal identification numbers, and from the municipality’s electronic patient record system from January 2017 to September 2018.

### Institutionalization and need of care

Data on institutionalization from baseline to the start of the re-examination in September 2018, and data on need for daily formal or informal care at time of the re-examination were gathered from the municipality’s electronic patient record system.

### Chronic conditions

In addition to the baseline information, data on the participants’ acquired chronic conditions before January 2017 were gathered from the official Finnish Care Register for Health Care including the Register of Primary Health Care Visits, and from the municipality’s electronic patient record system from baseline to re-examination. The chronic conditions and their 10th revision of the International Statistical Classification of Diseases and Related Health Problems (ICD–10) [20] considered in this study are shown in Additional file 1.

### Re-examination

The re-examination was performed in collaboration with the study nurse and the study physician at the Lieto Health Care Center or at the participants’ home. It included an extensive interview on socioeconomic factors, health behavior, physical ability, sense of well-being and quality of life. The interview included an assessment of the participants’ SRH, self-reported ability to walk 400 m with or without difficulties (referred to from now on as self-reported walking ability) and self-reported satisfaction with life. Subjective health was assessed with these three variables similarly as in our previous study: a person was considered subjectively healthy if they had good SRH, were self-reportedly able to walk 400 m and had good satisfaction with life [16].

Measurements of weight, height, blood pressure, pulse, vision and hearing were performed. Blood pressure values were recorded as the mean of two measurements. Also the Mini-Mental State Examination (MMSE) [21] and the Zung Self-Rating Depression Scale [22] were performed. Frailty by three frailty tools; FI, Frail Scale (FS) [23] and PRISMA-7 [24], was assessed. The same modifications to the FS and PRISMA-7 were used as in our previous studies [15, 17]. PBA was counted from the FI (for those with an FI of over 0) as previously described [12]. At baseline, the FI included 36 items, of which two missing items were allowed and the index was counted accordingly [15, 17]. At re-examination, the index consisted of 35 original items, of which also two missing items were allowed, and the index was counted accordingly (Additional file 2). PBA was compared to the CA of the same individual and their difference was counted. PBA was considered the same as CA if they were within a year of each other.

Physical functioning was measured by gait speed and grip strength. Gait speed was measured two times by the 4 m walking test when possible and substituted for the 2.5 m walking test in case of lack of space (if performed at the participants’ home). The fastest gait speed was recorded. Grip strength was measured two times using the dominant hand with the reliable and validated Jamar hydraulic hand dynamometer [25]. The highest grip strength was recorded.

The physical examination was performed by the study physician and it included a review of the participants’ medical records, including all medications used. Also, multiple laboratory tests were analyzed. Former diagnoses were recorded and new ones were set when appropriate. The participants were referred to additional examinations if necessary. The participants were all offered an extensive medical management plan and also the opportunity to discuss this plan and their own expectations for their future medical care with a nurse at the Lieto Health Care Center.

The eyeball test was based on the participants’ clinical appearance at time of the re-examination. The study physician assessed whether the participants seemed younger than their chronological age, the same, or older than their chronological age. The study physician made this judgment based on her clinical knowledge gathered caring for older people in primary care.

Data on successful agers was compared to the data of non-responders without daily informal or formal care. Additional analysis on life satisfaction was performed comparing the results of the successful agers with the re-examined participants with daily formal or informal care, and the non-responders with daily formal or informal care.

### Statistical analyses

The data acquired in the re-examination were combined with the data of the baseline examination to investigate the possible factors leading to SA. Differences between the variables at baseline and re-examination were examined with binary logistic regression for dichotomous variables and ordinal logistic regression for ordinal variables. Generalized estimating equations (GEE) with exchangeable correlation structure for dichotomous variables and independent correlation structure for ordinal variables were used to account for the correlation between the repeated measurements. Results are shown as odds ratios (OR) with 95% confidence intervals (CI). Sensitivity, specificity, positive predictive value and negative predictive value for the clinician’s eyeball assessment to identify participants with a PBA younger than their CA was calculated. Differences in categorical variables between the participants and the non-participants were examined with the χ^2^-test and difference in mean age with the two-sample *t* test. *P* values less than 0.05 were considered statistically significant. All statistical analyses were performed using SAS System for Windows, version 9.4 (SAS Institute Inc., Cary, NC, USA).

## Results

### Baseline characteristics

Baseline characteristics are shown in Table 1 together with follow-up characteristics. The participants’ mean age was 67.8 years (SD 2.5, range 64–77). Of the participants, 55% were women, 16% lived alone, only 1 % used mobility aids, over 90 % exercised weekly and had MMSE scores of 26 or over. Nine out of ten were able to self-reportedly walk 400 m and over 90 % reported their SRH as at least moderate. Satisfaction with life was high with only 3 % reporting poor satisfaction with life.

**Table 1:** Baseline and re-examination characteristics and change in the characteristics during the follow-up period

	Baseline (1998–99) *n =* 112	Re-examination (2018) *n =* 112	Re-examination vs. baseline (2018 vs. 1998–99)	
Age, years	mean (SD) [range]			
	67.8 (2.5) [64–77]	87.6 (2.5) [84–96]		
	n (%)		OR (95% CI)	*P*-value
Gender				
Men	50 (45)	50 (45)		
Women	62 (55)	62 (55)		
Living situation				
with someone	94 (84)	52 (46)	1	
alone	18 (16)	60 (54)	6.03 (3.69–9.83)	<.001
Mobility aid^a^	*n =* 111			
no	110 (99)	52 (46)	1	
yes	1 (1)	60 (54)	124.81 (17.39–895.54)	<.001
Exercise	*n =* 107			
weekly	99 (93)	81 (72)	1	
less than weekly/not at all	8 (7)	31 (28)	4.82 (2.20–10.55)	<.001
Self-reported walking ability	*n =* 110			
yes	100 (91)	84 (75)	1	
no	10 (9)	28 (25)	3.25 (1.51–7.01)	0.003
MMSE^b^		n = 111		
26–30	107 (96)	59 (53)	1	
≤ 25	5 (4)	52 (47)	18.68 (6.80–51.34)	<.001
Self-rated health^c^				
good	56 (50)	32 (29)	1	
moderate	51 (46)	57 (51)	1	
poor	5 (4)	23 (21)	2.93 (1.90–4.51)	<.001
Satisfaction with life^c^				
good	94 (84)	78 (70)	1	
moderate	15 (13)	33 (29)	1	
poor	3 (3)	1 (1)	2.28 (1.35–3.85)	0.002
Subjective health				
healthy	52 (46)	28 (25)	1	
not healthy	60 (54)	84 (75)	2.60 (1.61–4.20)	<.001
Number of chronic conditions				
0–2	100 (89)	41 (37)	1	
3–4	11 (10)	48 (43)	1	
≥ 5	1 (1)	23 (21)	14.87 (7.65–28.89)	<.001
Frail Scale^d^	*n =* 109	*n =* 109		
robust	90 (83)	44 (39)	1	
pre-frail	19 (17)	53 (49)	1	
frail	0 (0)	14 (13)	8.04 (4.75–13.50)	<.001
PRISMA-7	*n =* 110	*n =* 110		
robust	107 (97)	28 (25)	1	
frail	3 (3)	82 (75)	104.45 (31.94–341.61)	<.001
Frailty Index^d^	*n =* 107	*n =* 107		
robust	33 (30)	8 (7)	1	
pre-frail	73 (68)	50 (47)	1	
frail	2 (2)	49 (46)	12.88 (6.47–25.66)	<.001

*OR *Odds ratio; binary logistic regression using generalized estimating equations for dichotomous variables and ordinal logistic regression using generalized estimating equations for ordinal variables

^a^Having any mobility aid was compared to not having a mobility aid

^b^Mini-Mental State Examination

^c^Poorer category was compared to better categories

^d^Being frail was compared to being pre-frail or robust

Only 1 % had five or more chronic conditions, most had none to two. In addition, only three and 2 % were categorized as frail by PRISMA-7 and the FI, respectively, and none by the FS.

At baseline, the mean CA was 67.8 while the mean PBA was 61.3 years (Table 2).

**Table 2:** Chronological and personal biological age of the participants at baseline and re-examination, and their difference

	Baseline (1998–99)	Re-examination (2018)
Chronological age, years	*n =* 112	*n =* 112
mean (SD) [range]	67.8 (2.5) [64–77]	87.6 (2.5) [84–96]
Personal biological age, years	*n =* 105	*n =* 111
mean (SD) [range]	61.3 (11.5) [35.7–81.5]	77.1 (11.5) [36.3–94.7]
Difference of chronological and personal biological age, years	*n =* 105	*n =* 111
mean (SD) [range]	6.44 (11.21) [−16.47–32.88]	10.48 (11.01) [−8.25–48.70]

### Re-examination results and changes during the follow-up

The participants’ mean age was 87.6 years (SD 2.5, range 84–96). Most of them lived alone.

In all variables analyzed, there was a statistically significant change depicting poorer physical ability and subjective health at re-examination than at baseline. The use of a mobility aid had increased during the follow-up period. However, 75% of the participants were still self-reportedly able to walk 400 m, and 72% also still exercised weekly. The results for gait speed and grip strength are shown in Table 3.

**Table 3:** The results of the re-examination for gait speed and grip strength and the normative values

Results	Age, years	Men	Women	Normative values	Reference	Age, years	Men	Women	Remarks
		Mean (SD) [range]					Mean (SD)		
*Gait speed, m/s*		*n =* 50	*n =* 62		(Lusardi et al., 2003)	80–89	0.88 (0.24)	0.80 (0.20)	The number of participants in this age category was small
	≥84	1.06 (0.32) [0.36–2.00]	0.87 (0.30) [0.36–2.00]			≥90	0.72 (0.14)	0.71 (0.23)	The number of participants in this age category was small
					(Hollman et al., 2011)	80–85	1.12 (0.17)	1.01 (0.15)	Many common illnesses were considered as exclusion criteria in this study
						≥85	1.01 (0.22)		The number of participants in this age category was small and there were no women
*Grip strength,kg*		*n =* 50	*n =* 62		(Finnish Institute for Health and Welfare (THL), 2018)	≥80	32 (95% CI 30–34)	20 (95% CI 19–21)	The number of participants in these age categories were 108 for men and 184 for women
	≥84	32.0 (8.5) [12–51]	19.8 (5.4) [10–31]		(Werle et al., 2009)	80–84	31 (9)	19 (5)	The minimum size for each age category was 28 participants
						≥85	22 (6)	17 (5)	

*SD* Standard deviation

*CI* Confidence interval

Of the participants, 53% still had MMSE scores of 26 or over, although the proportion of participants with lower MMSE scores had increased significantly during the follow-up period. The most frequent number of chronic conditions was three to four, and 21% of the participants now had five or more chronic conditions.

Still 80% of the participants described their SRH as at least moderate. Also 99% of them were at least moderately satisfied with their lives and only one reported poor satisfaction with life. Some of the participants with good satisfaction with life at baseline had transitioned into the category of moderate satisfaction during the follow-up period, but the number of participants with poor satisfaction with life had decreased. However, of the participants, only 25% were categorized as comprehensively subjectively healthy (including having good SRH, being able to self-reportedly walk 400 m and having good satisfaction with life).

Frailty, by any tool used, had increased during the follow-up period. At re-examination, most of the participants were categorized as frail by PRISMA-7. By FS, most of them were pre-frail. By the FI, the proportions for frail and pre-frail were almost the same.

At re-examination, the mean CA was 87.6 years while the mean PBA was 77.1 years. The difference between the CA and the PBA was greater at re-examination than at baseline. There was one participant with an FI score of zero at re-examination so their PBA was not possible to count. With the logic of PBA, however, their PBA would have been substantially younger than their chronological age.

The concordance of the clinician’s eyeball assessment with the difference between the participants’ CA and PBA is shown in Table 4. Of the participants included in the analyses, no one was clinically assessed as seeming older than their chronological age, when in fact there were 12 participants with a PBA higher than their CA (three categorized as seeming younger than their CA and nine as seeming the same as their CA).

**Table 4:** Concordance of personal biological age and clinical assessment, the eyeball test

	Clinical assessment, the eyeball test^e^	
*n =* 110^a^	Younger than their chronological age	Same as their chronological age
Personal biological age in relation to chronological age	n	
Personal biological age younger than chronological age	68	25
Personal biological age same as^b^ or older than chronological age	4	13
Sensitivity (95% CI)^c^	0.73 (0.63–0.81)	
Specificity (95% CI)^c^	0.76 (0.53–0.90)	
Positive predictive value (95% CI)^d^	0.94 (0.87–0.98)	
Negative predictive value (95% CI)^d^	0.34 (0.21–0.50)	

*CI* Confidence Interval; Wilson method

^a^The clinical assessment was missing for one participant and in addition, for one participant, the personal biological age was not possible to count

^b^Personal biological age was considered the same as chronological age if they were within a year of each other

^c^Sensitivity and specificity of the eyeball test in identifying participants with a PBA younger than their CA from participants with PBA the same as CA or PBA older than CA

^d^Positive and negative predictive value for the eyeball test when identifying participants as seeming younger than their chronological age from participants with PBA the same as CA or PBA older than CA

^e^None of the participants were clinically assessed as seeming older than their chronological age

### Non-response analysis

There was no significant difference between the age of the participants and non-participants (Table 5). There was no significant difference in gender, living situation, SRH, self-reported walking ability or satisfaction with life between the non-participants and the participants. However, a larger proportion of the non-participants were in need of a mobility aid than the participants.

**Table 5:** Differences between the non-participants and participants

	Non-participants *n =* 44	Participants *n =* 112	*P* value
Age, years	mean, (SD) [range]		
	87.8 (2.6) [84–94]	87.6 (2.5) [84–96]	0.657
	n (%)		
Gender			0.346
Men	16 (36)	50 (46)	
Women	28 (64)	62 (55)	
Living situation	*n =* 43		0.788
Alone	22 (51)	60 (54)	
With someone	21 (49)	52 (46)	
Mobility aid	*n =* 43		0.008
Yes	33 (77)	60 (54)	
No	10 (23)	52 (46)	
Self-reported walking ability^a^			0.091
Yes	27 (61)	84 (75)	
No	17 (39)	28 (25)	
Self-rated health			0.220
Good	8 (18)	32 (29)	
Moderate	22 (50)	57 (51)	
Poor	14 (32)	23 (21)	
Satisfaction with life	*n =* 43		0.051
Good	24 (56)	78 (70)	
Moderate	16 (20)	33 (29)	
Poor	3 (7)	1 (1)	

^a^Self-reported ability to walk 400 m

A larger proportion of successful agers were satisfied with their lives on re-examination than the re-examined participants with daily formal or informal care or the non-responders with daily formal or informal care (Additional file 3).

## Discussion

Naturally, during 20 years, the physical and cognitive ability of the participants had decreased, but despite of that, most of them were satisfied with their lives.

More of them used mobility aids, were unable to walk 400 m, and had lower MMSE scores than at baseline. The participants exercising weekly were also expectedly fewer than at baseline. The results for the gait speed were in line with previous research for people over 85 years of age [26, 27], although these earlier studies had only a small group of participants in this age group. The average grip strengths were similar to the normative values found in a population study in Finland for people aged 80 years or over [28]. However, in our study, participants were all aged 84 years or over, and their results were better than in a Swiss population of same-aged participants [29].

The participants’ objective health, measured by the number of chronic conditions, had worsened during the follow-up period. However, only 21% of them had five or more chronic conditions, which in our previous study on the Lieto study population was associated with an increased risk of institutionalization [18]. Also, more of the participants assessed their SRH as poor, and less as good at the re-examination than at baseline. However, still over three fourths assessed their SRH as moderate or good. One explanation could be that the successful agers compare themselves to age-peers already deceased or institutionalized and therefore think they must be in good to moderate health.

For satisfaction with life, the proportion of participants with poor satisfaction had in fact decreased. Almost all of the participants considered their satisfaction with life as at least moderate despite that they had more illnesses, were more in need of walking aids, their MMSE scores were lower and they were more often frail than at baseline. This could suggest psychological resilience and the sensation of ageing successfully as even though their health was worse, they still had moderate or even good satisfaction with life. This finding is similar to the those of the Fordham Centenarian Study [6].

Only 25% of the participants were categorized as subjectively healthy at re-examination. At baseline, the proportion was 46%. The definition of good subjective health in this study was the same used in our previous study [16] where subjective health was found to have an additive effect on objective health in predicting mortality. The definition was, however, quite exclusive as being subjectively healthy required being self-reportedly able to walk 400 m, having a good SRH and a good satisfaction in life. At re-examination, the proportion of participants meeting each of these criteria had decreased leading by definition to also a decrease in the participants categorized as subjectively healthy. Still, one in four managed to meet all these three criteria at the chronological age of 84 years or over.

Frailty by all three frailty tools increased with ageing, which is in line with previous research [30]. As PRISMA-7 gives one point for all who are aged 85 years or over and a person requires three points in order to be categorized as frail, it was predictable that the number of participants categorized as frail by the PRISMA-7 would be high. PRISMA-7 is not therefore a very good frailty tool to be used on participants aged 85 years or older as it tends to over screen [31]. The FS categorized no one as frail at baseline and 13% at re-examination, less than the other tools. This finding is similar to our previous studies [15, 17] where we used the same modifications to the FS and PRISMA-7 and those could have affected the results.

Only 2 % of the participants were frail by the FI at baseline. However, despite that most of them were categorized as pre-frail, the mean PBA of the participants’ was lower than their mean CA at baseline. This persisted at re-examination. The omission of one of the original FI items unlikely impacted the results as the FI was counted accordingly.

The difference between the CA and the PBA was greater at re-examination than at baseline. By this assessment, they were indeed successful agers as the difference between the CA and the PBA continued to grow even during the long follow-up period of 20 years. PBA has earlier been found a better predictor of mortality than CA in a shorter follow-up of originally older study participants [12]. Our study supports that finding as these survivors were mostly biologically younger than their years. Despite more of them being frail at re-examination, they were not as frail as their chronological age would suggest and thus their risk of death was lower [32]. PBA evaluations are somewhat population specific because of the way they are counted and direct comparisons between populations should be made with caution [12].

The concordance of the PBA and the eyeball test was poor in finding the frail participants, the ones with a higher PBA than their CA, a similar result to earlier research [14]. The eyeball test as earlier been shown to have poor inter-rater reliability, at least when done by non-geriatricians [33].

However, when identifying the successful agers, the participants with a PBA lower than their CA, the eyeball test had a good positive predictive value.

The strengths of our study are a long follow-up, extensive data gathered at baseline and at re-examination including a thorough clinical assessment and measures of physical fitness, and the multiple data sources used to gather information on physical ability, morbidity, mortality, institutionalization and use of formal or informal care. At re-examination, there was only one participant with missing data on MMSE score. The comparisons between the baseline and re-examination results were conducted without that one result. Also, for some of the re-examined participants, all data for frailty tools was not available at baseline and for that, they could not be included in comparisons made between the baseline and re-examination, although the data for the frailty tools were available at re-examination. The robust data at re-examination is a strength to this study. The obvious weakness to our study is the smallish sample size and a substantial number of the non-participants, which is often the case when studying the oldest population [34]. However, we managed to get some data on also the non-participants by mail, which can be considered as a strength to this study [34], and there were no significant differences between the participants and non-participants in relation to age, gender, living situation, SRH, self-reported walking ability or satisfaction in life. Despite this, there seems to be non-response bias, a common challenge in ageing research [35, 36], as there was a difference in physical ability between the non-participants and the participants. As the non-participants were more frequent users of mobility aids and as such supposedly in poorer physical condition, the inclusion of their results when measuring gait speed and grip strength could have diluted the good mean results now gathered without them. The additional analyses on life satisfaction comparing the successful agers with the re-examined participants with daily formal or informal care, and the non-responders with daily formal or informal care showed a statistically significant difference in favor of the successful agers. However, the numbers of the participants in the other categories were low.

## Conclusions

The participants were biologically younger than their chronological age, and satisfied with their lives both at baseline and at re-examination. Further research is required to determine possible causality between these factors and successful ageing. We aim to investigate these successful agers further by comparing them to non-survivors of the same age at baseline. We want to investigate the possible association of a lower PBA than CA, and good satisfaction with life with successful ageing. In addition, we aim to analyze the association of traditional risk factors such as hypertension, smoking and high cholesterol, with ageing successfully.

## Supplementary Information

**Additional file 1.**

**Additional file 2.**

**Additional file 3.**

## Data Availability

The datasets generated during and/or analyzed during the current study are available from the corresponding author on reasonable request.

## Abbreviations

CA: Chronological age
CI: Confidence interval
GEE: Generalized estimating eqs.
FI: Frailty index
FS: Frailty scale
ICD-10: 10th revision of the international Statistical classification of diseases and related health problems
MMSE: Mini-mental state examination
OR: Odds ratio
PBA: Personal biological age
SA: Successful ageing
SD: Standard deviation
SRH: Self-Rated health
