# Supplementary material for: Re-examination of successful agers with lower biological than chronological age still after a 20-year follow-up period
Source: BMC Geriatr. 2023 Mar 7;23:128. doi: 10.1186/s12877-023-03844-y (PMC9990196; doi:10.1186/s12877-023-03844-y)
Supplement: Supplementary file 1 — Additional file 1. [file 12877_2023_3844_MOESM1_ESM.pdf]

Appendix 1. Chronic conditions and their ICD-10[a] codes considered in this study

Chronic conditions	ICD-10
Malignant neoplasms (except basal cell carcinomas)	C0–C97 (except C44.01, C44.11, C44.21, C44.31, C44.41, C44.51, C44.61, C44.71, C44.81, C44.91)
Iron deficiency anaemia	D50
B12-vitamin anaemia	D51
Hypothyroidism	E03, E89
Diabetes mellitus	E10–E14
Hypercholesterolaemia	E78
Dementia	F00–F03, G30
Mood disorders	F30–F39
Systemic atrophies, extrapyramidal and movement disorders	G10–G26
Hypertension	I10–I15
Ischemic heart disease	I20–I25
Atrial fibrillation	I48
Intracranial haemorrhage	I60–I62
Stroke	I63–I69, G45
Atherosclerosis	I70
Chronic lower respiratory diseases	J40–J47
Renal failure	N17–N19

[a]10th revision of the International Statistical Classification of Diseases and Related Health Problems
