# Supplementary material for: Re-examination of successful agers with lower biological than chronological age still after a 20-year follow-up period
Source: BMC Geriatr. 2023 Mar 7;23:128. doi: 10.1186/s12877-023-03844-y (PMC9990196; doi:10.1186/s12877-023-03844-y)
Supplement: Supplementary file 2 — Additional file 2. [file 12877_2023_3844_MOESM2_ESM.pdf]

Appendix 2. Items included in the Frailty Index at re-examination

Needs help with toileting	Angina pectoris
Needs help with dressing and undressing	Other medical problems[b]
Needs help with preparing meals	No regular physical exercise
Needs help with house work	Vision problem
Needs help with heavy household chores	Hearing problem
Needs help with personal care	Feeling hopeless
Needs help with moving about inside house	Emotional problem
Arthritis or rheumatism	Memory problem
High blood pressure	Bodily pain
Chronic bronchitis or emphysema	Speech problem
Diabetes mellitus	Resting tremor
Heart disease[a]	Five or more medications
Cancer	Difficulties carrying or lifting light loads
Stomach or intestinal ulcers	Mobility problem
Suffers from the effect of stroke	Limited kind of amount of activity
Urinary incontinence	Feeling tired all the time
Stool incontinence	Weight loss
Hip or femoral fracture	

[a]Known heart disease (ICD-10: I20–I25, I48, I49)
[b]Other disease (ICD-10: E03, E05, G20, G35, J44–J46, M15–M17, M47)
