# Supplementary material for: Re-examination of successful agers with lower biological than chronological age still after a 20-year follow-up period
Source: BMC Geriatr. 2023 Mar 7;23:128. doi: 10.1186/s12877-023-03844-y (PMC9990196; doi:10.1186/s12877-023-03844-y)
Supplement: Supplementary file 3 — Additional file 3. [file 12877_2023_3844_MOESM3_ESM.pdf]

Additional file 3. Satisfaction with life in successful agers, re-examined participants with daily formal or informal care and non-participants with daily formal or informal care

	Successful agers n=112	Re-examined participants with daily care n=26	Non-participants with daily care n=11
		n (%)	
Satisfaction with life			
Good	78 (70)	13 (50)	4 (36)
Moderate	33 (30)	11 (42)	4 (36)
Poor	1 (1)	2 (8)	3 (27)

Successful agers compared to re-examined participants with daily care $p=0.03$
Successful agers compared to non-participants with daily care $p<.001$
